# Differential roles of tryptophan residues in conformational stability of *Porphyromonas gingivalis* HmuY hemophore

**DOI:** 10.1186/1471-2091-15-2

**Published:** 2014-02-10

**Authors:** Marcin Bielecki, Halina Wójtowicz, Teresa Olczak

**Affiliations:** 1 Laboratory of Biochemistry, Faculty of Biotechnology, University of Wroclaw, F. Joliot-Curie 14A St., 50-383 Wroclaw, Poland

**Keywords:** Heme, Hemophore, HmuY, *Porphyromonas gingivalis*, Protein unfolding

## Abstract

**Background:**

We have previously shown that the *P. gingivalis* HmuY hemophore-like protein binds heme and scavenges heme from host hemoproteins to further deliver it to the cognate heme receptor HmuR. The aim of this study was to characterize structural features of HmuY variants in the presence and absence of heme with respect to roles of tryptophan residues in conformational stability.

**Results:**

HmuY possesses tryptophan residues at positions 51 and 73, which are conserved in HmuY homologs present in a variety of bacteria, and a tryptophan residue at position 161, which has been found only in HmuY identified in *P. gingivalis* strains. We expressed and purified the wildtype HmuY and its protein variants with single tryptophan residues replaced by alanine or tyrosine residues. All HmuY variants were subjected to thermal denaturation and fluorescence spectroscopy analyses. Replacement of the most buried W161 only moderately affects protein stability. The most profound effect of the lack of a large hydrophobic side chain in respect to thermal stability is observed for W73. Also replacement of the W51 exposed on the surface results in the greatest loss of protein stability and even the large aromatic side chain of a tyrosine residue has little potential to substitute this tryptophan residue. Heme binding leads to different exposure of the tryptophan residue at position 51 to the surface of the protein. Differences in structural stability of HmuY variants suggest the change of the tertiary structure of the protein upon heme binding.

**Conclusions:**

Here we demonstrate differential roles of tryptophan residues in the protein conformational stability. We also propose different conformations of apo- and holoHmuY caused by tertiary changes which allow heme binding to the protein.

## Background

Periodontitis is an infectious disease in which genetic, microbial, immunological, and environmental factors combine to influence disease risk and progression, resulting in the destruction of tooth-supporting tissues [1,2]. There is growing evidence showing that a number of complex human diseases are caused or are influenced by periodontal diseases, including cardiovascular and respiratory diseases, diabetes mellitus, osteoporosis, and rheumatoid arthritis [3,4]. A major etiological agent in the development and progression of chronic periodontitis is *Porphyromonas gingivalis*, a black-pigmented Gram-negative anaerobic bacterium [1].

Establishment of infection by pathogens depends on a readily available iron, and for some bacteria a heme source. However, iron and heme availability is limited in the host environment and therefore bacteria evolved sophisticated systems to acquire these compounds from host hemoproteins. Among them, Gram-negative bacteria utilize outer-membrane receptors directly or with the assistance of a hemophore [5-9]. Unlike other Gram-negative bacteria, *P. gingivalis* does not produce and utilize siderophores. The bacterium also lacks the majority of enzymes of the biosynthetic pathway for heme biosynthesis. Therefore, *P. gingivalis* must acquire heme as a sole source of iron and protoporphyrin IX. In *P. gingivalis*, several TonB-dependent outer-membrane receptors for heme utilization have been described, including HmuR, Tlr, IhtA, and HusB [5,8]. The best characterized system of heme acquisition in *P. gingivalis* consists of proteins encoded by *hmu* operon, comprising the HmuR and HmuY proteins. HmuR is an outer-membrane TonB-dependent receptor involved in heme transport through the outer membrane [10-13], whereas HmuY is a membrane-associated heme-binding lipoprotein [14-16]. We reported that HmuY binds iron(III) protoporphyrin IX and iron(II) protoporphyrin IX at 1:1 molar ratio [14,15]. Detailed characterization of the HmuY-heme complex demonstrated that heme, with a midpoint potential of 136 mV, is in a low-spin Fe(III) hexa-coordinate environment [17]. In that report we identified histidine residues at positions 134 and 166 as potential heme ligands. Crystallographic analysis determined unique beta-fold HmuY protein structure and confirmed engagement of H134 and H166 in heme binding [18]. We also demonstrated that HmuY binds iron(III) mesoporphyrin IX and iron(III) deuteroporphyrin IX in an analogous way to heme [19]. The only differences observed were two forms of HmuY-iron(III) deuteroporphyrin IX complex differing by a 180° rotation of porphyrin around the *α-γ-meso*-carbon axis. Recently we showed that HmuY forms complexes with non-iron metalloporphyrins, though recognition of the ligand by HmuY depends on the central metal ion [20]. Among them, Ga(III)PPIX, Co(III)PPIX, and Cu(II)PPIX exhibited antimicrobial activity against *P. gingivalis*[21]. Similar to hemophores, HmuY was demonstrated to wrest heme from methemoglobin [9,22]. Heme extraction from oxyhemoglobin was facilitated after oxidation to methemoglobin by pre-treatment with *P. gingivalis* R-gingipain A (HRgpA) and K-gingipain (Kgp) [9] or *Prevotella intermedia* interpain A [22]. Heme uptake served by HmuR receptor and hemophore-like HmuY lipoprotein is novel and has been identified for the first time in *P. gingivalis*.

In this work we report structural features of HmuY variants and the protein’s conformational changes induced by temperature and studied by means of fluorescence spectroscopy. Studies presented in this work have been performed using the wildtype HmuY protein and its variants with single tryptophan residues replaced by alanine or tyrosine residues. Due to the widespread location of tryptophan residues in HmuY, one could expect that observation of tryptophan fluorescence spectra could help to explain local structural stability of the protein and conformational changes accompanying ligand binding.

## Methods

### Site-directed mutagenesis and protein purification

The DNA sequence encoding *P. gingivalis* HmuY polypeptide part lacking the first 25 residues (NCBI accession no. CAM 31898) and cloned into pHmuY11 plasmid [15] served as a template for mutagenesis. Single tryptophan residues were replaced by alanine or tyrosine residues using QuikChange II XL Site-Directed Mutagenesis Kit (Stratagene). Primers designed and used in this study are listed in Table 1. All HmuY protein variants were overexpressed using respective plasmids and *Escherichia coli* ER2566 cells (New England Biolabs), and purified from a soluble fraction of *E. coli* lysates as previously described [16]. Molecular modeling of HmuY protein variants was performed with the GROMOS96 implementation of Swiss-PdbViewer (version 4.1).

**Table 1:** Primers designed and used in this study

**Construct**	**Mutation**	**Template**	**Primer name**	**Primer sequence**
HY11W51A	W51A	pHmuY11	HY11W51AF	5’- cgatgcttcgaaatacgaaacgGCgcagtatttctctttttccaaagg-3’
			HY11W51AR	5’- cctttggaaaaagagaaatactgcGCcgtttcgtatttcgaagcatcg-3’
HY11W73A	W73A	pHmuY11	HY11W73AF	5’- ctataagaacgatttgaacGCGgacatggctcttca-3’
			HY11W73AR	5’- gcggtgaagagccatgtcCGCgttcaaatcgttcttatag-3’
HY11W161A	W161A	pHmuY11	HY11W161AF	5’- agggatttgcttcaggtggtGCGctggaattctctcacggtc-3’
			HY11W161AR	5’- gaccgtgagagaattccagCGCaccacctgaagcaaatccct-3’
HY11W51Y	W51Y	pHmuY11	HY11W51YF	5’- cgatgcttcgaaatacgaaacgTATcagtatttctctttttccaaagg-3’
			HY11W51YR	5’- cctttggaaaaagagaaatactgATAcgtttcgtatttcgaagcatcg-3’
HY11W73Y	W73Y	pHmuY11	HY11W73YF	5’- ctataagaacgatttgaacTATgacatggctcttcaccgc-3’
			HY11W73YR	5’- gcggtgaagagccatgtcATAgttcaaatcgttcttatag-3’
HY11W161Y	W161Y	pHmuY11	HY11W161YF	5’- agggatttgcttcaggtggtTATctggaattctctc-3’
			HY11W161YR	5’- gaccgtgagagaattccagATAaccacctgaagcaaatccct-3’

### Preparation of holoproteins

Hemin (Hemin chloride; ICN Biomedicals) solutions were prepared and UV-visible titration experiments were carried out as described previously [17]. Holoforms of all HmuY variants were formed by incubating apoprotein with heme in the molar ratio 1:1.2. Excess heme was separated by gel filtration using a PD-10 desalting column (Amersham Pharmacia) or Zeba Spin Desalting Columns (Thermo Scientific). HmuY concentration was determined spectrophotometrically using the empirical extinction coefficients ε_280_ = 36.68 mM^-1^ cm^-1^ and ε_280_ = 59.26 mM^-1^ cm^-1^ for apo- and holoform, respectively, determined as reported previously [17,20]. Extinction coefficients of HmuY variants constructed in this study were calculated analogously using the following equation:

$$\varepsilon_{280N}=\varepsilon_{280D}\left(\frac{AN_{280}}{AD_{280}}\right)$$

where *ε280D* is the theoretical extinction coefficient (http://www.expasy.org) whilst *AN280* and *AD280* are absorbancies of native and unfolded protein in 20 mM phosphate buffer pH 7.4, containing 20 mM NaCl and the same buffer containing 6 M guanidine hydrochloride (Gdn-HCl; MP Biomedicals), respectively. The empirical extinction coefficients are as follows: ε_280_ = 32.16, 27.75, 27.66, 30.82, 29.37, 28.45 mM^-1^ cm^-1^ for W51A, W73A, W161A, W51Y, W73Y, W161Y, respectively. Titration curves were analyzed using equations for a one-site binding model and *K*_d_ values determined as reported earlier [19] using the OriginPro 8 software (OriginPro Corporation).

### Circular dichroism (CD) spectroscopy

Far-UV CD spectroscopy was carried out using a Jasco J-810 spectropolarimeter. Protein samples at 2 μM concentration in 20 mM sodium phosphate buffer, pH 7.4, were measured in a 10-mm path-length cell. The spectra were recorded over a wavelength range of 190–260 nm by signal averaging of four spectra. Scanning speed of 100 nm/min and step resolution of 1 nm were used. All spectra were baseline corrected for respective buffers.

### Intrinsic tryptophan fluorescence spectroscopy

Intrinsic tryptophan fluorescence emission spectra were recorded using a Jasco FP750 spectrofluorometer. Protein samples at 4 μM concentration in 20 mM sodium phosphate buffer, pH 7.4, containing 20 mM NaCl were excited at 295 nm and emission spectra were recorded in the range of 300–450 nm, at a scanning interval of 1 nm and integration time of 1 s. All spectra were baseline corrected for respective buffers. For analysis of heme binding, 4 μM protein samples in 20 mM sodium phosphate buffer, pH 7.4, containing 20 mM NaCl were titrated with 1-μl aliquots of 1 mM hemin prepared as described previously [17]. Fluorescence was determined at excitation at 295 nm and emission at 329 nm.

### Thermal denaturation

Unfolding experiments were performed according to standard protocols with modifications described previously [18]. Changes in intrinsic tryptophan fluorescence emission spectra due to thermally induced protein unfolding were monitored at 315 nm for excitation at 295 nm using a Jasco FP750 spectrofluorometer. Protein samples at concentration of 4 μM in 20 mM sodium phosphate buffer, pH 6.5, containing 20 mM NaCl and 1 M GdnHCl [18] were heated (22°C–85°C) in 10-mm path-length cells with time/temperature interval of 1°C per minute.

Sypro Orange (SYPRO ORANGE protein gel stain, Invitrogen) exhibits intensive fluorescence when bound to hydrophobic residues of proteins and therefore it was used as a probe for thermal unfolding of HmuY. Samples of 40 μM proteins in 20 mM sodium phosphate buffer, pH 7.4, containing 20 mM NaCl were placed in a 96-well qPCR plate and Sypro Orange was then added according to the manufacturer’s instructions (Invitrogen). After 1-h equilibration at 25°C the plate was heated up to 80°C in an Mx3005P thermocycler (Agilent Technologies) with a rate of 0.5°C per 30 s. Fluorescence was monitored by a single photomultiplier tube (PMT) detector using a FROX filter (excitation wavelength 585 nm, emission wavelength 610 nm). Fluorescence data were transformed to yield the relative fraction of unfolded protein [18].

## Results and discussion

**Figure 1:**
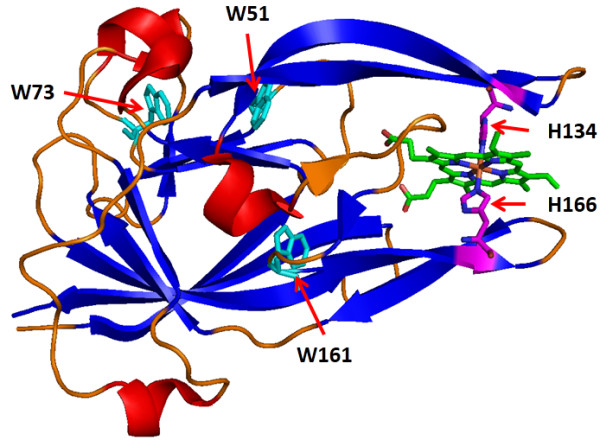
**Structure of heme-bound HmuY (PDB code: 3H8T).** Stick representation of tryptophan side chains with a defined color for each structure is shown. α-helices are marked in red, β-strands in blue, loops in orange, tryptophan residues in cyan, and axial histidine residues in purple.

*P. gingivalis* HmuY possesses three tryptophan residues at positions 51, 73 and 161 [18], evenly distributed in the polypeptide chain and protein structure; W161 is buried in the interior of the heme binding cavity whilst W73 and W51 are located closer to the protein surface at the loops between β-strands forming the central twisted β-sandwich structure of the holoHmuY (Figure 1). Furthermore, W51 is partially exposed on the holoHmuY surface just at the joint of the protein core and a protruding β-hairpin, forming a heme binding site that contains H134, one of the iron coordinating histidines [17,18]. Due to the widespread location of tryptophan residues one could expect that observation of tryptophan fluorescence spectra can help to explain local structural stability of the protein and conformational changes accompanying ligand binding. Therefore we constructed protein variants of the HmuY protein with single tryptophan residues replaced either by alanine or tyrosine residues (namely W51A or W51Y, W73A or W73Y, and W161A or W161Y). In this study, we attempted to obtain a maximum effect of lacking a tryptophan side chain on structural stability of the protein by replacing it with an alanine residue. In contrast, tyrosine substitution was expected to preserve the minimal free energy of the holoHmuY native conformation. Even though subtle structural changes were imposed by some aminoacid replacements, as observed from far-UV CD spectra (Additional file 1: Figure S1), they did not significantly affect HmuY function since all protein variants preserved highly similar affinity to heme binding compared to the wildtype protein, as examined by fluorescence analysis (Additional file 2: Figure S2) and determination of heme dissociation constants (0.216 ± 0.007 μM, 0.238 ± 0.012 μM, 0.235 ± 0.011 μM for W51Y, W73Y, W161Y, respectively and 0.303 ± 0.013 μM, 0.229 ± 0.008 μM, 0.323 ± 0.016 μM for W51A, W73A, W161A, respectively, compared with 0.296 ± 0.012 μM for the wildtype HmuY).

A tryptophan in a hydrophobic environment exhibits a maximum wavelength of the fluorescence emission spectra at 320–335 nm, while in a hydrophilic or exposed environment it exhibits a maximum at 340–360 nm [23]. Therefore intrinsic fluorescence emission, by selective excitation of tryptophan residues, was used to probe local conformational fluctuations in HmuY. When excited at 295 nm, all proteins show rather wide fluorescence emission spectra. The wildtype HmuY and all but the W51Y protein variants show maxima within a wavelength range from 328 to 330 nm. However, tyrosine substitution of the apo-form of W51 results in slight blue shift of the spectrum maximum to 325 nm. It is again in good agreement with W51 being exposed to the more polar environment. Interestingly, W51A mutant has a maximum at 328 nm, which may result from structural differences of the protein variant imposed by the lack of a large aromatic side chain on the protein surface that normally would make the structure more rigid, protecting it from penetration by solvent molecules.

**Figure 2:**
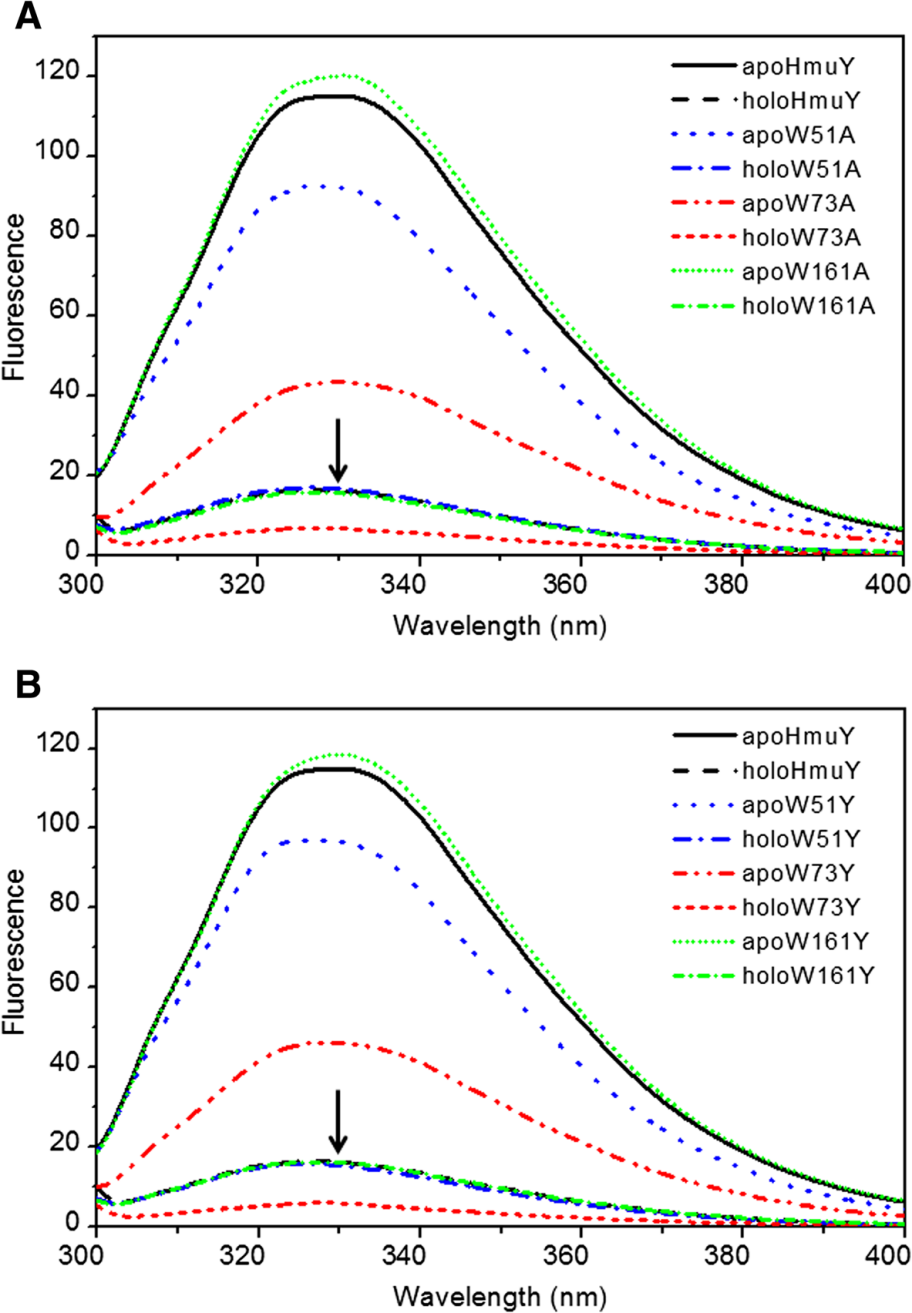
**Intrinsic tryptophan fluorescence spectra of apoform and holoform of HmuY alanine-substituted (A) and tyrosine-substituted (B) protein variants.** Emission spectra of 4 μM protein samples in 20 mM sodium phosphate buffer, pH 7.4, containing 20 mM NaCl were recorded between 300 and 450 nm at excitation at 295 nm. Overlapping curves marked with arrows correspond to (A) holoHmuY, holoW51A, and holoW161A or (B) holoHmuY, holoW51Y, and holoW161Y. Representative data out of three independent experiments are shown.

Heme binding results in quenching of HmuY intrinsic fluorescence and holoHmuY retains about 14% of the fluorescence intensity of the apoform (Figure 2). Spectra of the wildtype holoHmuY and substituted holoW51 or holoW161 are comparable. In contrast, W73A and W73Y protein variants reflect only about 5% of initial apoHmuY intensity after heme binding. It is noteworthy that replacement of W73 has a different effect on the apo- and holoform of the protein. (Figure 2), pointing to W73 fluorescence being quenched more severely in the HmuY-heme complex with greater exposure to the hydrophilic exterior of holoHmuY in comparison to apoHmuY.

While W161 is the most buried tryptophan residue in the protein core its impact on fluorescence spectra of native protein is marginal. Both alanine and tyrosine W161 variants have almost identical fluorescence spectra as the wildtype HmuY (Figure 2), which implicates that W161 residue is completely quenched. This may be due to aromatic ring π stacking between W161 and proximal F194. In addition, high energy states of W161 indoile group can be discharged by formation of the hydrogen bond between side ring nitrogen of W161 and the main chain carbonyl oxygen of Y80. Similarity in spectra of apoHmuY and holoHmuY as well as of W161 variants suggest that both hydrogen bond and tryptophan and phenylalanine ring arrangements remain the same in both protein forms.

Substitution of W51 or W73 by either a tyrosine or an alanine residue results in loss of about 18% or 60% of apoHmuY fluorescence intensity (Figure 2). W51 and W73 residues are located in close proximity to each other and in holoHmuY, as can be seen in the crystal structure [18], W51 being the most exposed tryptohpan residue to a more hydrophilic environment on the protein surface. Therefore energy from W73 can be transferred to W51 and then quenched due to the polar environment of the latter. This also explains why fluorescence spectra of each protein variant do not sum to the spectra of the wildtype protein. Since W161 seems not to contribute to the spectra of apoHmuY, W51A, W73A, W51Y and W73Y can serve as representatives for protein variants where only fluorescence emission spectra of W73 or W51, respectively, can be observed. We assumed that spectra of W73A and W73Y simply represent fluorescence of W51 whilst spectra of W51A and W51Y correspond to fluorescence intensity of W73 when none of its energy is transferred to W51. Thus, it has been useful for observation of HmuY thermal unfolding and concluding conformational changes accompanying ligand binding.

**Figure 3:**
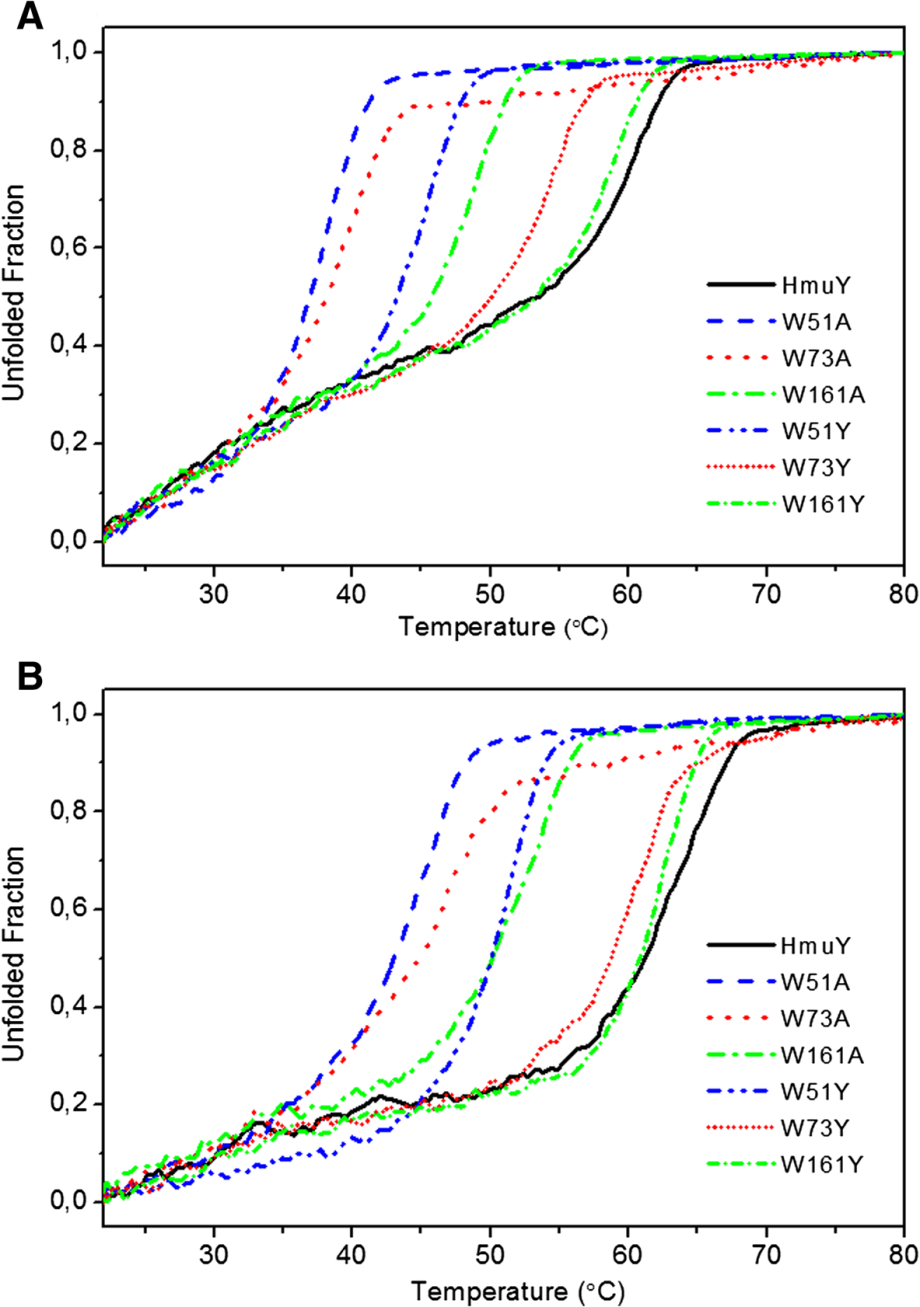
**Normalized equilibrium curves of apoform (A) and holoform (B) of HmuY protein variants.** Denaturation was carried out using 4 μM protein samples in 20 mM sodium phosphate buffer, pH 6.5, containing 20 mM NaCl and 1 M GdnHCl. Samples ware heated in 10-mm path-length cells with a rate of 1°C per min. Spectra ware monitored with excitation at 295 nm and emission at 315 nm. Representative data out of three independent experiments performed in triplicate are shown.

Our predictions were well reflected in the experimental observations from HmuY thermal unfolding experiments and alanine substitutions of tryptophan residues generally resulted in greater destabilization of HmuY structure compared with tyrosine substitutions (Figure 3). The most profound effect of the lack of a large hydrophobic side chain was observed for W73 as the difference in T_m_ between apoW73Y (54.11 ± 0.051°C) and apoW73A (39.45 ± 0.055°C) was 15°C (Figure 3A). The same differences for protein variants with affected W51Y (45.21 ± 0.031°C) or W51A (37.64 ± 0.043°C) and W161Y (58.81 ± 0.066°C) or W161A (48.81 ± 0.039°C) were 8°C and 10°C, respectively (Figure 3A). This tendency was preserved for heme-loaded proteins (Figure 3B): W73Y (60.11 ± 0.079°C) or W73A (46.09 ± 0.10°C), W51Y (50.53 ± 0.04°C) or W51A (44.41 ± 0.057°C), W161Y (62.12 ± 0.054°C) or W161A (52.12 ± 0.06°C). In all cases, replacement of the W51 exposed on the surface resulted in significant loss of protein stability and even the large aromatic side chain of a tyrosine residue had little potential to substitute this tryptophan residue. In contrast, replacement of the most buried W161 with a tyrosine residue only moderately affects protein stability: apoW161Y (58.81 ± 0.066°C) or holoW161Y (62.12 ± 0.054°C) versus apoHmuY (60.38 ± 0.095°C) or holoHmuY (63.45 ± 0.11°C). Alanine substitution of W161 showed a greater effect on HmuY stability, but W161A still remained the most stable of all alanine-substituted variants (48.81 ± 0.039°C and 52.13 ± 0.062°C, for apo- and holoprotein, respectively). It is worth noting that W51 and W73 of HmuY, compared with W161, which is unique for *P. gingivalis*, are conserved among homologous HmuY protein sequences found in other bacteria [16,24]. We suspect that they may be important for conformational stability of HmuY protein and participate in tertiary changes allowing heme binding.

**Figure 4:**
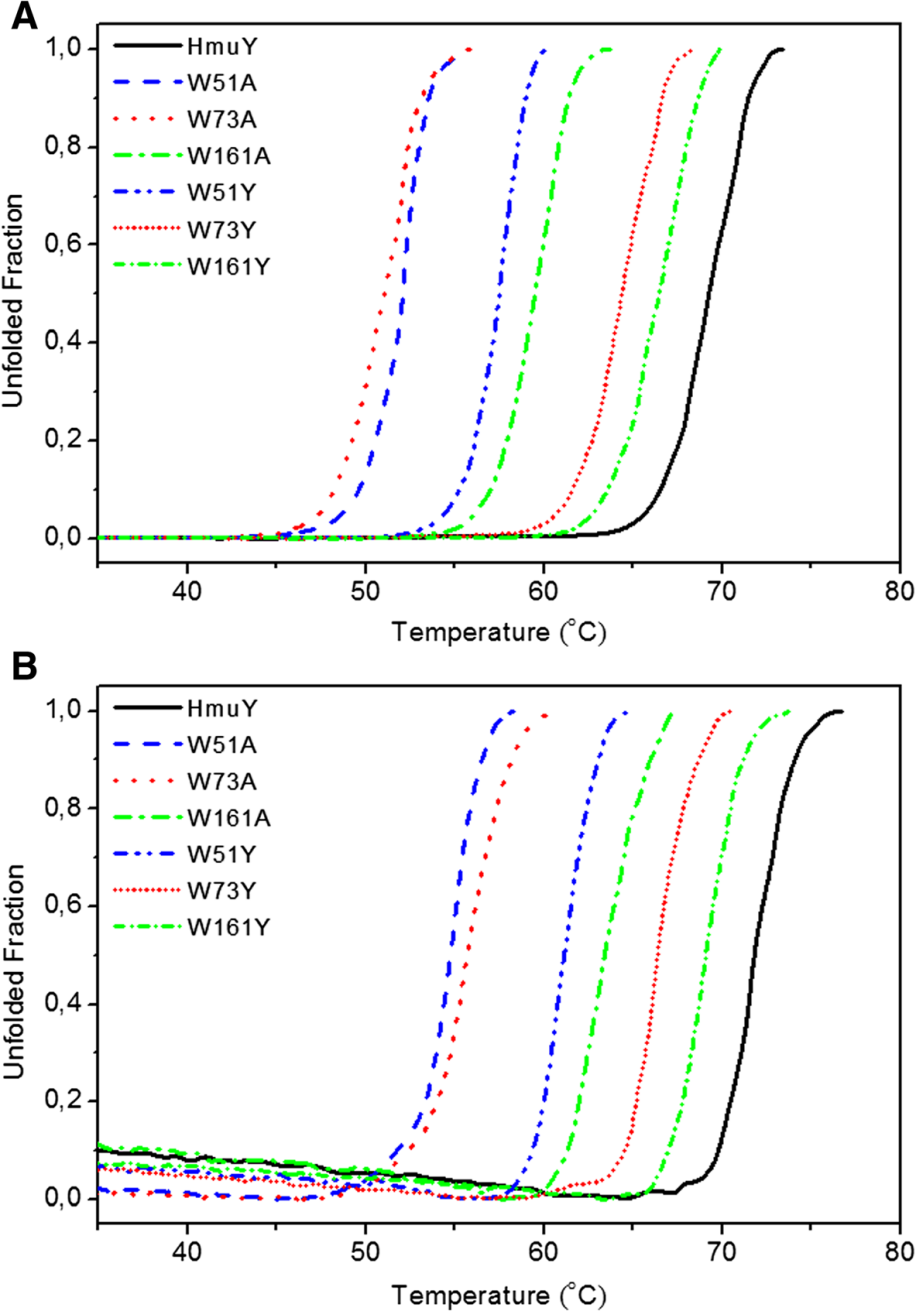
**Normalized equilibrium curves of apoform (A) and holoform (B) of HmuY protein variants.** Protein samples at concentration 40 μM in 20 mM sodium phosphate buffer, pH 7.4, containing 20 mM NaCl and Sypro Orange were heated from 25°C to 80°C with a rate of 0.5°C per 30 s. Probe fluorescence was monitored with excitation at 585 nm and emission at 610 nm. Representative data out of three independent experiments performed in triplicate are shown.

Thermal unfolding of HmuY protein variants was further monitored using Sypro Orange fluorescence (Figure 4). Interestingly, apoW161Y mutant unfolds analogously to the wildtype apoHmuY (66.82 ± 0.48°C versus 69.55 ± 0.31°C) (Figure 4A). Heme binding raises W73Y stability to the level of the wildtype apoprotein (Figure 4B). Lack of compensation of the destabilization introduced by replacement of the W51 residue by ligand binding emphasizes the key role of this residue in preserving protein folding. In general, higher T_m_ values were observed for processes monitored with Sypro Orange probe. Discrepancies between melting temperatures obtained using different techniques may result from different phenomena being observed. Sypro Orange binds to the denaturing protein and offers the possibility to observe general exposure of hydrophobic side chains to the solvent whilst changes in intrinsic protein emission fluorescence are sensitive to local environment distortions around selected tryptophan residues. One may also not exclude possibility that differences observed may result from different buffers used. Previously, we reported that thermal denaturation of HmuY in 20 mM sodium phosphate buffer, containing 20 mM NaCl was irreversible, leading to protein precipitation [18], but addition of 1 M GdnHCl resulted in a reversible process. Under these conditions apo- and holoHmuY preserved their native conformations and holo-HmuY retained bound heme [18], thus demonstrating that under the concentration used GdnHCl did not cause protein denaturation and allowed analysis of HmuY stability using intrinsic fluorescence. To analyze general denaturation process using SyproOrange fluorescence, 1 M GdnHCl was omitted because it interferes with the probe.

Finally, it should be noted that HmuY may function as monomer and, especially after heme binding, may form dimers and tetramers [15,18]. Therefore, influence of oligomerization on protein stability and heme transfer should be taken into consideration. However, in this study we assumed that under conditions used (protein concentration at 4–40 μM) HmuY is present in solutions in the form of a monomer. Such assessment was based on our preliminary, unpublished data (*i.e.* results from gel filtration, analytical ultracentrifugation and small angle X-ray scattering analysis). These data suggest that HmuY oligomerization may occur at higher protein and salt concentration [18].

Based on our data, one may expect conformational changes occurring during heme binding to HmuY and probably during heme release from HmuY. We propose that HmuY first binds free heme or preferably wrests heme bound to hemoproteins, mainly hemoglobin, and then releases it for subsequent binding to HmuR. The initial step in heme unbinding may involve disruption of only one of the two axial histidine ligands of HmuY. Our previous studies showed that single replacement of H134 or H166 by an alanine residue did not result in abolishing heme binding, which at least in part may support this hypothesis. This reversible intramolecular coordination by a histidine chain may allow heme transfer from hemoproteins to HmuY and subsequently to HmuR, similar to typical bacterial hemophores. It has been shown that the hemophore HasA from *Serratia marcescens*[25,26] or *Pseudomonas aeruginosa*[27,28] exhibits large rearrangements, allowing heme binding to H32 and Y75. However, a recent study demonstrated that H32 is not conserved in all secreted hemophores. For example, HasA from *Yersinia pestis* coordinates heme with a single Y75 and its structure in both apo- and holoform is almost identical [29]. Thus, analysis of heme binding to HmuY is of importance in understaning heme transfer into the *P. gingivalis* cell.

## Conclusions

In this study we demonstrate differential roles of tryptophan residues played in the HmuY conformational stability. We also hypothesize that the changes observed may result from different tertiary structure of apo- and holoHmuY. At least so far, we were unable to solve the three-dimensional structure of apoHmuY, allowing detailed comparison of apo- and holoforms. Therefore results gained in this study may at least in part support our hypothesis of tertiary changes allowing heme binding to *P. gingivalis* HmuY.

## Competing interests

The authors declare that they have no competing interests.

## Authors’ contributions

MB, HW, TO conceived and designed the experiments, MB, HW performed the experiments, HW, MB, TO analyzed the data, TO, HW, MB wrote the paper. All authors read and approved the final manuscript.

## Authors’ information

Marcin Bielecki and Halina Wojtowicz share the first authorship.

## Supplementary Material

Additional file 1: Figure S1 Far-UV circular dichroism (CD) spectra measured for apoHmuY (A) and holoHmuY (B) alanine variants or apoHmuY (C) and holoHmuY (D) tyrosine variants. Protein samples at 2 μM concentration in 20 mM sodium phosphate buffer, pH 7.4 were analyzed in a 10-mm path-length cell. The spectra were recorded over a wavelength range of 190–260 nm. Representative data out of three independent experiments with similar tendency are shown. Click here for file

Additional file 2: Figure S2 Heme titration of HmuY alanine (A) and tyrosine (B) variants. Heme binding was monitored by quenching of intrinsic tryptophan fluorescence. Protein samples at 4 μM in 20 mM sodium phosphate buffer, pH 7.4, containing 20 mM NaCl were analyzed in 10 mm quartz cuvette. The spectra were recorded from 300 to 450 nm at excitation at 295 nm. Representative data out of three independent experiments with similar tendency are shown. Click here for file
