# Supplementary material for: Differential roles of tryptophan residues in conformational stability of Porphyromonas gingivalis HmuY hemophore
Source: BMC Biochem. 2014 Feb 10;15:2. doi: 10.1186/1471-2091-15-2 (PMC3922309; doi:10.1186/1471-2091-15-2)
Supplement: Additional file 1: Figure S1 — Far-UV circular dichroism (CD) spectra measured for apoHmuY (A) and holoHmuY (B) alanine variants or apoHmuY (C) and holoHmuY (D) tyrosine variants. Protein samples at 2 μM concentration in 20 mM sodium phosphate buffer, pH 7.4 were analyzed in a 10-mm path-length cell. The spectra were recorded over a wavelength range of 190–260 nm. Representative data out of three independent experiments with similar tendency are shown. [file 1471-2091-15-2-S1.docx]

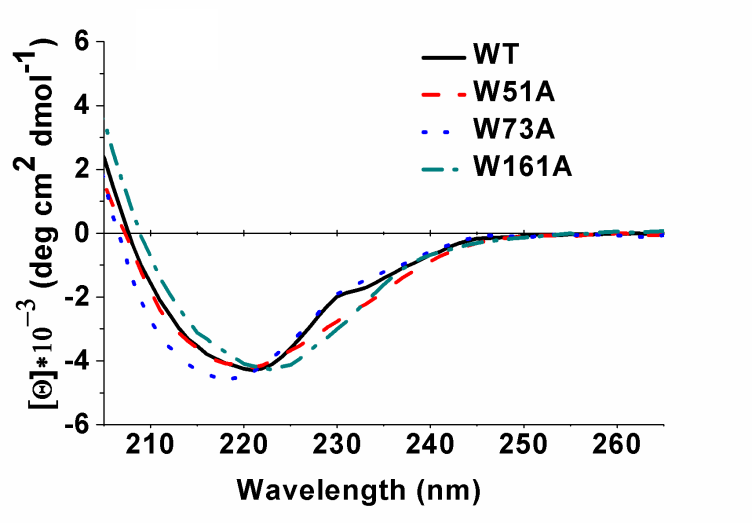

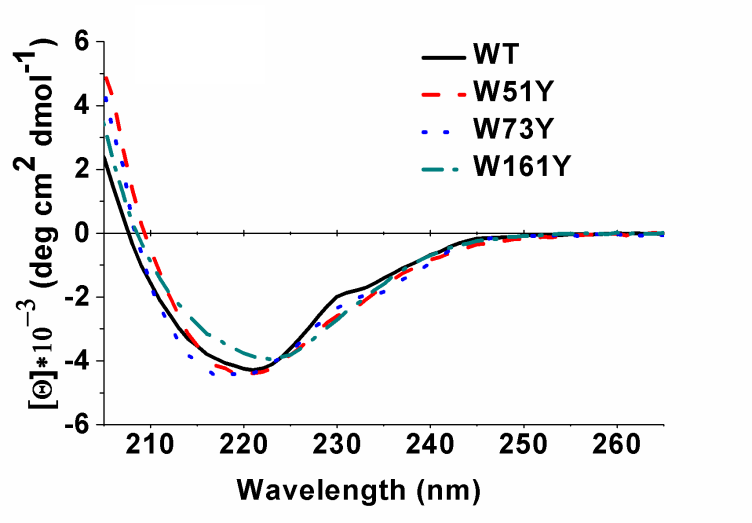

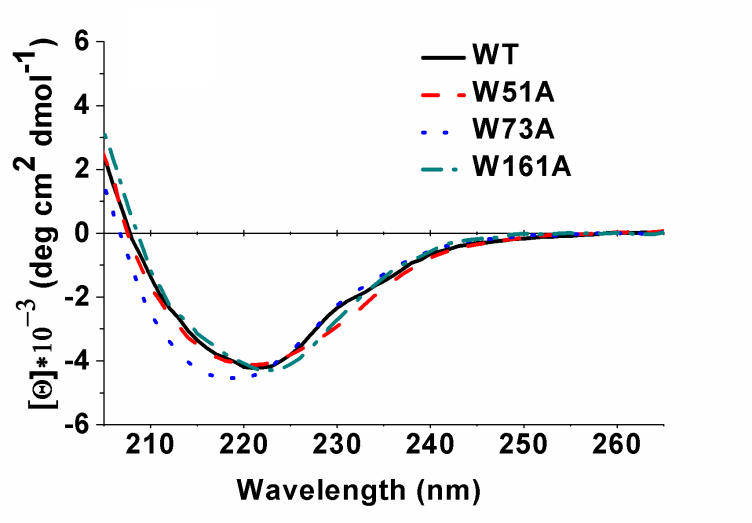

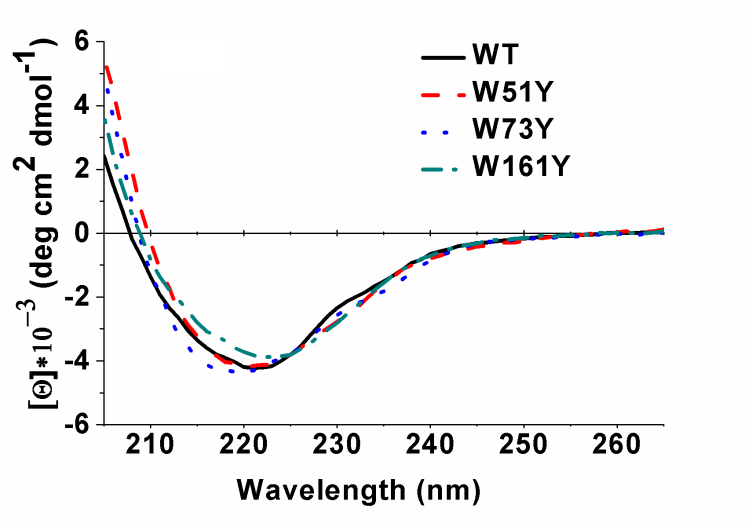


**C**

**A**

**B**

**D**

**Figure S1. Far-UV circular dichroism (CD) spectra measured for apoHmuY (A) and holoHmuY (B) alanine variants or apoHmuY (C) and holoHmuY (D) tyrosine variants.** Protein samples at 2 µM concentration in 20 mM sodium phosphate buffer, pH 7.4 were analyzed in a 10-mm path-length cell. The spectra were recorded over a wavelength range of 190-260 nm. Representative data out of three independent experiments with similar tendency are shown.
