# Supplementary material for: Differential roles of tryptophan residues in conformational stability of Porphyromonas gingivalis HmuY hemophore
Source: BMC Biochem. 2014 Feb 10;15:2. doi: 10.1186/1471-2091-15-2 (PMC3922309; doi:10.1186/1471-2091-15-2)
Supplement: Additional file 2: Figure S2 — Heme titration of HmuY alanine (A) and tyrosine (B) variants. Heme binding was monitored by quenching of intrinsic tryptophan fluorescence. Protein samples at 4 μM in 20 mM sodium phosphate buffer, pH 7.4, containing 20 mM NaCl were analyzed in 10 mm quartz cuvette. The spectra were recorded from 300 to 450 nm at excitation at 295 nm. Representative data out of three independent experiments with similar tendency are shown. [file 1471-2091-15-2-S2.docx]

**B**

**A**

**Figure S2. Heme titration of HmuY alanine (A) and tyrosine (B) variants.** Heme binding was monitored by quenching of intrinsic tryptophan fluorescence. Protein samples at 4 µM in 20 mM sodium phosphate buffer, pH 7.4, containing 20 mM NaCl were analyzed in 10 mm quartz cuvette. The spectra were recorded from 300 to 450 nm at excitation at 295 nm. Representative data out of three independent experiments with similar tendency are shown.
